# Sequential activation of the AKT pathway in human osteoblasts treated with Oscarvit: a bioactive product with positive effect both on skeletal pain and mineralization in osteoblasts

**DOI:** 10.1186/s12891-017-1860-2

**Published:** 2017-11-28

**Authors:** Tibor Görögh, Elgar S. Quabius, Alexander Georgitsis, Markus Hoffmann, Sebastian Lippross

**Affiliations:** 1 Department of Otorhinolaryngology, Head and Neck Surgery, Christian-Albrechts-University Kiel, Germany, Arnold-Heller-Str. 3 House 27, 24105 Kiel, Germany; 2 Institute of Immunology, Christian-Albrechts-University Kiel, Germany, Arnold-Heller-Str. 3 House 17, 24105 Kiel, Germany; 3 Department of Orthopaedic Trauma Surgery, Christian-Albrechts-University Kiel, Germany, Arnold-Heller-Str. 3 House 18, 24105 Kiel, Germany

**Keywords:** Oscarvit, Osteoporosis, Osteoblast, Gene expression, Mineralization

## Abstract

**Background:**

Oscarvit (OSC) is an in-house preparation consisting of calcium, magnesium, phosphorus, strontium, Vitamin D, and eggshell membrane hydrolysate containing naturally occurring glycosaminoglycans and sulfated glycoproteins. OSC has been used both in an open-label human study and in vitro in osteoblasts.

**Methods:**

Fifteen patients divided into three groups received oral OSC 0.6 g three times daily for 20 days. The main outcome measures were regional skeletal pain over the treatment period. For the in vitro experiments eight primary human osteoblasts cultures were established from trabecular bone, six of them from the femoral head, and two from the tibia. Cells were cultured for five to 20 days in the presence of 20 μg/ml OSC. Immunocytochemistry and RT-PCR were used to detect molecular alterations involved in the mineralization process. Calcium concentration was measured by means of a colorimetric assay and cell viability was analyzed using the LDH cytotoxicity assay. To investigate whether the osteoblasts response to OSC is associated with signaling processes the ERK1/2 and AKT signal transduction pathways were analyzed.

**Results:**

Open label human study: OSC, 0.6 g three times daily, resulted in a significant positive effect on pain alleviation of 42% after 5 days (*p* < 0.001), 57% after 10 days and 68% after 20 days (*p* < 0.0001; for both time points), with no side-effects being reported. In vitro analysis: In osteoblasts, growing in OSC-supplemented media significant overexpression of bone γ-carboxylglutamic acid-containing protein, secreted phosphoprotein-1, integrin binding sialoprotein, and dentin matrix phosphoprotein genes could be detected when compared to control osteoblasts grown in the absence of OSC. Moreover, OSC-treated osteoblasts produced over the study period vast extracellular calcium deposits without any loss of cellular integrity or signs of cellular toxicity. In addition OSC promotes osteoblast differentiation and activates the AKT signaling pathway.

**Conclusion:**

This open label study provides preliminary evidence of the efficacy of OSC. Despite the limitations (small heterogeneous patient group) the findings can be viewed as a necessary investigation that supports further clinical trials with a double-blind controlled design. Experiments at cellular and molecular level provide supplementary information about OSC that increases mineralization in osteoblasts and activation of the AKT pathway.

**Trial registration:**

DRKS00013233, 06th November 2017, retrospectively registered.

## Background

Bone and connective tissue disorders are often considered a local problem centered on the specific target area where bone degradation is seen**.** With the aging of the world’s population the incidence of osteoporosis and related skeletal diseases increases constantly. Recent developments in the treatment of bone atrophy and the prevention of osteoporotic fractures have provided modern agents that inhibit bone resorption and lead to increased bone density i.e. specific antibodies or strontium ranelate [1, 2]. In search for the appropriate treatment of these diseases also bioactive natural products are increasingly used with favorable effect on bone metabolism [3]. Nevertheless numerous alternative preparations (e.g. Norzoanthamine [4], Phorbaketal A [5], Fucoxanthin [6]) are commercially available but lack scientific evidence of in vivo effectiveness.

Here, we present the use of OSC in a single-center open-label human study as a possible natural therapeutic for skeletal disorders. Several aspects of OSC’s possible action can be assumed when the ingredients are separately looked at. For example, strontium ranelate is a new class of osteoporosis medication, capable of accelerating bone formation and, to a certain extent, inhibiting bone resorption [2, 7, 8]. Calcium and vitamin D are important dietary supplements to prevent osteoporosis, and several other minerals amongst them iron, copper, and selenium, are known to play a role in the prevention of osteoporosis [9]. Eggshell membrane is a natural source of glycosaminoglycans such as chondroitin, glucosamine, and sulfated glycoproteins [10, 11]. Glucosamine and chondroitin sulfate are able to protect subchondral bone [12, 13] shown in an animal model of osteoporosis [14]. Chondroitin was also shown to act on subchondral bone osteoblasts by modulating the osteoprotegerin/receptor activator of NF-kB ligand ratio in favor of reduced bone resorption [15].

In addition to the open-label study, we also analyze the effect of OSC on cellular and molecular level. As a primary target we established osteoblast cultures from femoral head specimens. Human osteoblasts are commonly used to analyze bone metabolism, osteogenesis and focal bone erosion in patients with joint diseases. The most widely used differentiation marker for these cells is alkaline phosphatase production. In contrast, cells devoid of osteoinductive ability show lower alkaline phosphatase activity [16]. The molecular analyses performed here should provide information on the expression of genes coding for integrin binding sialoprotein (IBSP), bone γ-carboxylglutamic acid-containing protein, (BGLAP), secreted phosphoprotein-1, (SPP1), and dentin matrix acidic phosphoprotein (DMP1) all belonging to the most abundant non-collagenous bone matrix proteins expressed in osteoblasts [17, 18]. Although their precise mechanism of action is not always understood, these non-collagenous bone matrix proteins play an important role in bone mineralization in part by their ability to react with the mineral components of the bone [19]. To show whether osteoblast response to OSC is associated with signal transduction events, we also analyze the ERK1/2, and AKT signal transduction pathways as essential mediators in diverse biological responses such as cell differentiation, mineralization, and proliferation of various cell types including osteoblasts [20, 21].

The data presented here show the results obtained from the open-label human study, and describes the responses of human osteoblasts to OSC.

## Methods

### Patients and osteoblast cultures

We studied 15 patients (12 female, three male) with osteoporosis of the low back (group 1; *n* = 2) of the low back and hip (group 2; *n* = 4), and both osteoporosis and osteoarthritis of the hip (group 3; *n* = 9). All diagnoses were confirmed by iDXA and X-ray. Mean age of the patients was 69 years (range: 57 to 83 years). All patients were informed about the OSC treatment and gave written informed consent to the participation in this open-label study. A precondition for participating in the study was that, prior to the OSC-treatment, the patients had not taken medications which could have a significant influence on the course of the study. In addition the patients had to have pain and discomfort in the diseased areas at rest and during physical activity. Overweight patients (weighing more than 100 kg) were excluded from the study. The patients received three times daily 0.6 g OSC orally in vegetarian capsules, over a period of 20 days. The dosage was based on in vitro to in vivo extrapolation [22] of the amount of OSC used in our cell mineralization experiments. Pain level was assessed by a 0–10 Numeric Pain Rating Scale (NPRS) where patients rated their pain on a score where 0 = no pain; 5 = moderate pain; 10 = worst possible pain. Pain intensity was evaluated 5, 10, and 20 days after starting the OSC-treatment.

Eight primary human osteoblast cultures were established from trabecular bone tissue from osteoporotic patients (aged between 62 and 93 years). Six cultures were established from the femoral head, and two from the tibia. The bone specimens were briefly rinsed in PBS, cut into small pieces (1–2 mm in diameter), and then thoroughly rinsed in PBS. The small bone fragments were gently shaken with 2 mL crude collagenase (Biochrom, Berlin, Germany) at 37 °C for 2 h and then rinsed in PBS. The isolated cells were incubated in minimum essential medium (MEM) with 10% (*v*/v) fetal calf serum, 100 U/mL penicillin/streptomycin (Biochrom) at 37 °C in 5% CO_2_ humidified atmosphere and were left for 4 days before the first medium change. When primary cultures were confluent cells were detached with trypsin/EDTA (Biochrom) and pelleted. The cells were reconstituted in MEM and counted in a hemocytometer to ensure that an equal number of cells were seeded in the various experimental cultures. Dermal fibroblasts grown in MEM under identical conditions served as control cells.

### Scanning electron microscopy (SEM)

Osteoblasts were cultured on small glass discs and at required time-points fixed in 1.5% glutaraldehyde for 30 min at 4 °C, post-fixed in 1% osmium tetroxide for 1 h at 4 °C, and dehydrated through a series of increasing concentrations of ethanol and dried using hexamethyldisilazane (HMDS). Samples were sputter coated with gold using an ion coater (Balzers Union) and analyzed using a scanning electron microscope (Philips, Holland) at 10 kV.

### Alkaline phosphatase activity

Activity of alkaline phosphatase in osteoblasts was detected as previously described [23] using 5-bromo-4-chloro-3-indolyl-phosphate/nitro blue tetrazolium (BCIP/NBT) (0.35 mM BCIP, 0.37 mM NBT, 5 mM MgCl_2_, 100 mM Tris buffer, pH 9.5, 45 min) as a precipitating substrate according to the manufacturer’s protocol (Sigma-Aldrich). In brief, prefixed cells were rinsed with 0.1 M Tris-HCl buffer (pH 7.6) and incubated with substrate to obtain optimal staining intensity. After washing with Tris-HCl buffer, coverslips were embedded in antifading solution (glycerol gelatin; Sigma-Aldrich).

### Immunocytochemistry

After washing the cells with PBS they were fixed in alcohol and incubated each with primary mouse monoclonal anti-human collagen type-1 and osteocalcin antibody (Thermo-Scientific, Darmstadt, Germany) for 30 min at room temperature. Then they were incubated with biotinylated secondary goat anti-mouse antibody (Dako, Hamburg) for 30 min at room temperature in the dark and subsequently treated with avidin-biotin complex (Biozol, Eching, Germany) for 30 min. Negative controls were obtained by omission of the primary antibody. Cells were counterstained with hemalaun and immunocytochemical staining was assessed using a Zeiss microscopy (Axioplan, Jena, Germany).

### RT-PCR analysis

Total RNA of normal and test cells was extracted using the RNeasy Kit (Qiagen, Hilden, Germany) and cDNA synthesis was performed using MMLV reverse transcriptase following the manufacturer’s instruction (Life Technologies, Inc.). For RT-PCR analysis, the cDNA was amplified for 30 cycles using the sense 5′-CCAGCCGAGCCACATCGC-3′ and anti-sense 5′-ATGAGCCCCAGCCTTCTCCAT-3′ oligonucleotides specific for the GAPDH gene (annealing temperature 55 °C), the sense 5′-GATGACGATGAAGATGACAG-3′ and anti-sense 5′-CTCTTCACTCTCACTCTCTTG-3′ specific for the DMP1 gene (annealing temperature 58 °C), the sense 5′-GCAGCGAGGTAGTGAAGAG-3’ and anti-sense 5′-CGATGTGGTCAGCCAACTC-3′ specific for the BGLAP gene (annealing temperature 60 °C), the sense 5′-GCCATGACCACATGGATGAT-3’ and anti-sense 5′-GTCTACTGTGGGGACAACTG-3’ specific for the SPP1 gene (annealing temperature 63 °C), and the sense 5′-GAGATGACAGTTCAGAAGAG-3’and anti-sense 5′-TCATCCACTTCTGCTTCGC-3’ specific for the IBSP gene (annealing temperature 57 °C). The reverse transcription PCR products were analyzed by 1.2% agarose gel electrophoresis. Alpha Imager Gel Doc gel documentation software (Biozym, Germany) was used for quantitation of mRNA expression normalized by GAPDH mRNA expression.

### Osteoblast mineralization experiment

The test cells were cultivated under the same temperature and CO_2_ conditions as the control cells but the minimum essential medium (MEM) was supplemented with OSC (20 μg/ml), consisting of mineral components similar to those of the bone (8 μg/ml = 0.2 mM calcium, 0.016 μg/ml = 0.5 μM phosphorus, 0.08 μg/ml = 3 μM magnesium, 3 ng/ml = 30 nM strontium, 0.2 ng/ml = 3 nM iron, 0.1 ng/ml = 1 nM copper, and 0.2 pg/ml = 0.002 nM selenium, as well as 0.6 ng/ml = 0.02 IU Vitamin D and 2 μg/ml eggshell membrane hydrolysate containing glycosaminoglycans such as chondroitin, glucosamine, and sulfated glycoproteins [10]. The in vitro mineralization experiments were monitored over a period of 5, 10, 15, and 20 days respectively. The OSC doses and incubation times were determined in preliminary experiments (unpublished). Culture medium of both the test and the control cells supplemented with and without OSC was changed every second day. Calcium deposits in mineralized cultures were detected by staining with 10% Alizarin Red solution (Sigma-Aldrich) as described elsewhere [24]**.**

### Quantification of total calcium content

Control and test cells (2 × 10^6^ each) were grown over a period of 5, 10, 15, and 20 days in dish culture, rinsed twice with PBS and scraped off from the dishes using a cell scraper (Sarstedt, Nümbrecht, Germany). After centrifugation the cells were re-suspended and dissolved in 1 N HCl. Calcium content was measured using the Abcam Calcium Detection Assay Kit ab102505 (Abcam, Berlin). This colorimetric endpoint assay measures the amount of calcium–cresolphthalein complexone formed when cresolphthalein complexone binds to free calcium in alkaline solution within the physiological range of 0.1 mM–3.0 mM. Absorbance was measured at a wavelength of 575 nm with background subtraction of 490 nm using a microplate reader (Dynatech MR5000, Denkendorf, Germany).

### Cell cytotoxicity assay

Cell viability was measured using the non-radioactive LDH Detection Kit (Roche Diagnostics, Germany). Cells (2 × 10^6^ each) grown to monolayers were incubated for 15 days in MEM supplemented with OSC (for more information see induction of mineralization in Material Methods section). After centrifugation at 250 g for 10 min. The cell-free culture supernatants were collected and incubated according to the manufacturer’s instruction. To calculate cell viability day 15 control cells (2 × 10^6^) growing in MEM in absence of OSC were treated with Triton-×100 (1% *v*/v) for one hour and served as second control. Absorbance was measured at 492 and 620 nm using a 96-well plate ELISA reader (Dynatech MR5000, Denkendorf, Germany).

### ERK and AKT signaling pathways

Primary osteoblasts (2 × 10^6^ each) growing in MEM were either treated with 100 ng/ml IGF-1 (Life Technology, Darmstadt Germany) for 1 h, or cultivated in MEM supplemented with OSC (20 μg/ml) over a period of 1, 5, 10, and 15 days. The cells were harvested by centrifugation (800 g for 5 min) washed with PBS, and lysed in a solution containing 20 mM Tris/HCl (pH 7.4), 150 mM NaCl, 2 mM EDTA, 1% Igepal 0,5% SDS, 1 mM PMSF, 5% 2-mercaptoethanol, 10% glycerol in presence of protease inhibitor (Roche Diagnistics, Mannheim, Germany). Total protein extracts (10 μg) were analyzed by sodium dodecyl sulfate polyacrylamide gel electrophoresis (SDS-PAGE) and Western blot. The primary mouse monoclonal antibodies against AKT^PSer473^ and ERK1/2^PTyr204^ were purchased from Life Technology (Darmstadt, Germany) and the horse radish peroxidase-conjugated second antibody from Dako (Hamburg, Germany). Immune complexes were detected utilizing the Amersham ECL Chemi-Lumi detection system and Amersham Hyperfilm MP (GE Healthcare, Buckinghamshire, UK). The Alpha Imager Gel Doc gel documentation software (Biozym, Germany) was used for quantitation of protein expression.

### Statistical analysis

Data concerning the pain score evaluation were analyzed by means of repeat measure One-Way ANOVA, followed by Bonferroni post-testing (SPSS vs 20). Data were considered statistically significant if *p* < 0.05. All data are presented as mean values ± standard deviation (SD). All in vitro experiments were carried out in triplicate and analysis of variance was performed by means of One-Way ANOVA, followed by Bonferroni post-testing (SPSS vs 20).

## Results

### Open-label trial

In total 15 patients with osteoporosis of the low back (group-1; *n* = 2), of the low back and hip (group-2; *n* = 4), and both osteoporosis and osteoarthritis of the hip (group-3; *n* = 9), participated in a 20 day open-label trial of OSC. Characteristics of the study participants are presented in Table 1. In group-1 after 5-day, 10-day, and 20-day OSC-treatment, the pain level (assessed by a 0–10 NPRS), amounted to 7.5 leading to a percentage alleviation of pain (AP) of 25%, 6.0, AP = 40%, and 5.0, AP = 50%, and in group-2, 4.5, AP = 55%; 3.0, AP = 70%; and 2.5, AP = 75%, respectively (due to the small number of participants in groups 1 and 2 no statistical evaluation was possible). The average NPRS-values in group-3 were 6.0, AP = 40% (*p* = 0.001); 4.5, AP = 55% (*p* < 0.0001); and 3.1, AP = 69% (*p* < 0.0001), respectively. Considering all three groups together there was a statistical significant positive effect on pain alleviation of 42% after 5 days (*p* < 0.001), 57% after 10 and 68% after 20 days (*p* < 0.0001). OSC-treatment was well tolerated without any side effects.

**Table 1:** Characteristics of the study participants

	Sex				Mean NPRS^**a**^	
Pain region	Female	Male	Mean age (y)	5d	10d	20d
Low back	2	0	73	7.5	6.0	5.0
Low back and hip	3	1	67	4.5	3.0	2.5
Hip	7	2	66	6.0	4.5	3.1

^a^Numeric Pain Rating Scale. The patients rated their pain score after 5, 10, and 20 days by making

a cross on a score 0 = no pain; 5 = moderate pain; 10 = worst possible pain

### Characterization of osteoblasts

**Fig. 1:**
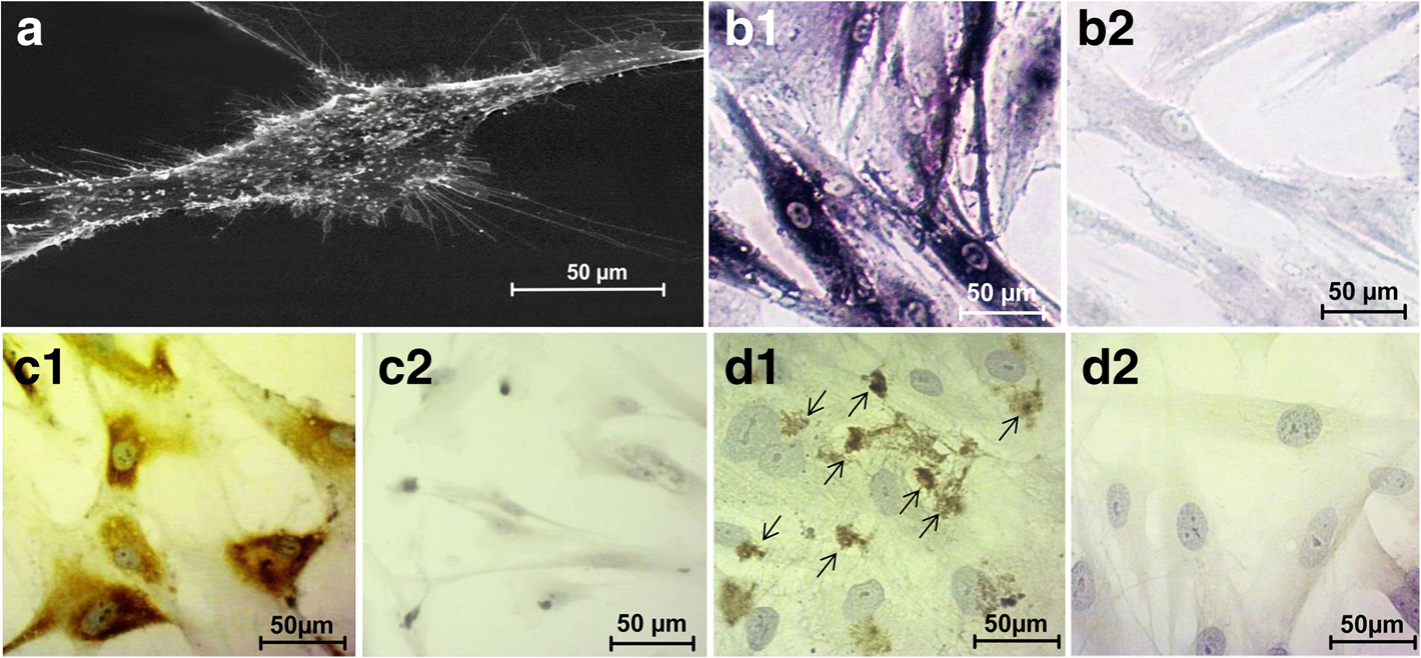
Morphological and immunocytochemical characteristics of osteoblasts from a primary culture. **a** Scanning electron micrograph demonstrating the architecture of a flattened fibroblast-like osteoblast with several fiber-like extensions. Strong accumulation of alkaline phosphatase (**b1**) and collagen type-1 (**c1**) are exhibited in human osteoblasts cells in contrast to normal fibroblasts (**b2** and **c2**). Osteoblasts demonstrate expression of BGLAP (arrows) (**d1**), whereas no immunoreactivity of this marker protein was detectable in normal dermal fibroblasts (**d2**)

Examination of primary osteoblast cells with scanning electron microscope (SEM) shows flattened three-dimensional fibroblast-like morphology on the smooth growth surface with numerous irregularly formed cell extensions (Fig. 1A). Osteoblasts demonstrate high alkaline phosphatase activity and synthesize collagen type-1 and BGLAP (Fig. 1 B1, C1, D1). The BCIP/NBT product to detect alkaline phosphatase was accumulated diffusely in the cytoplasm. Immunostaining for collagen type-1 was identified with near-uniform intensity in the cytoplasm, lacking or only partly present in apical cell regions after incubation with the conjugated monoclonal antibody. Under similar treatment conditions dermal fibroblasts expressed only a small amount of alkaline phosphatase and immunostaining for collagen type-1 and BGLAP was not detectable (Fig. 1 B2, C2, D2).

### Effect of OSC on gene expression

**Fig. 2:**
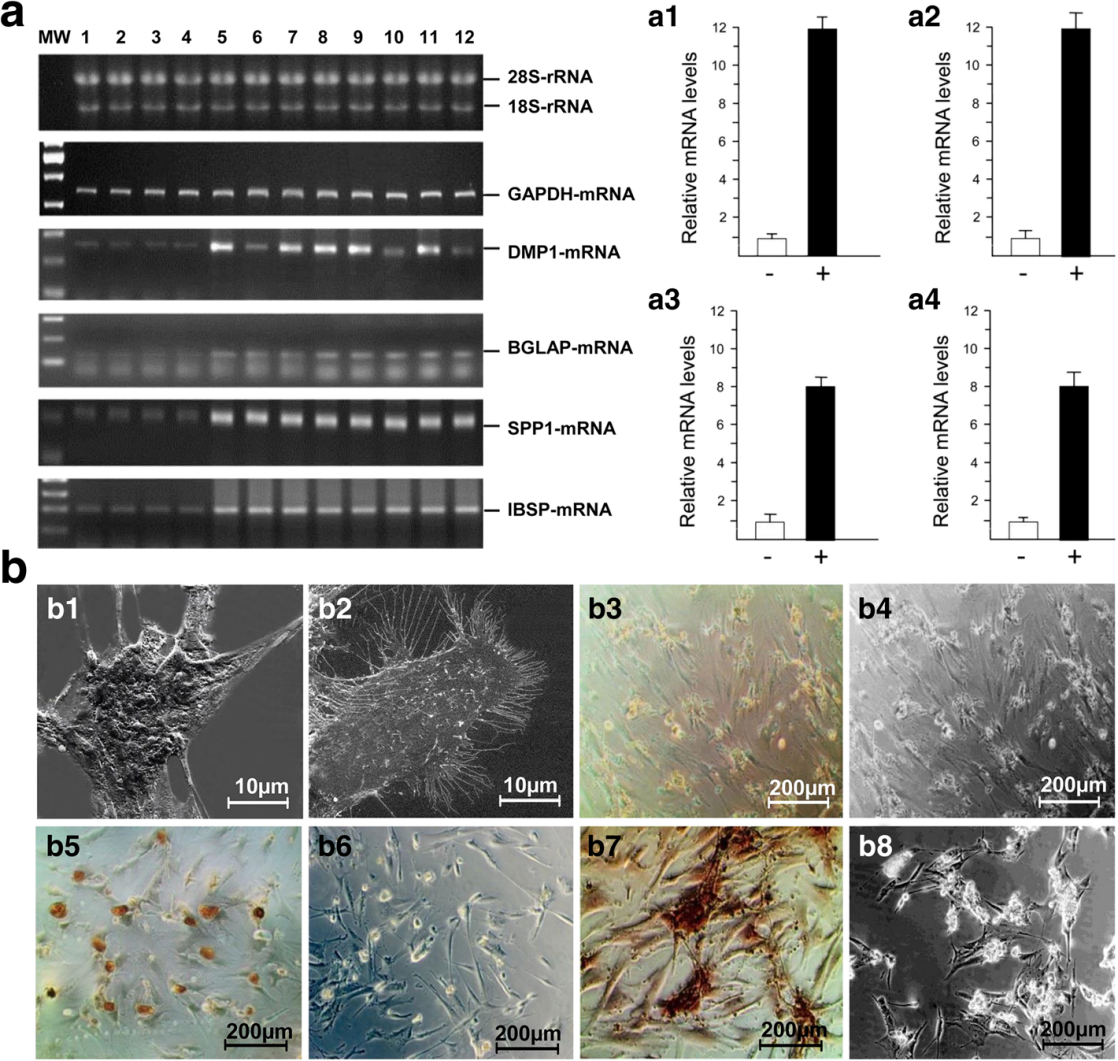
Analysis of osteoblast mineralization. **a** Agarose gel electropherograms showing the RT-PCR banding patterns of DMP1-, BGLAP-, SPP1- and IBSP-mRNA in osteoclasts growing in MEM without OSC (lanes 1–4) and with 20 μg/ml OSC (lanes 5–12) over a cultivation period of 15 days. Expression of the housekeeping gene GAPDH was used as a loading control. (**a1**-**a4**) Densitographic evaluations of the electropherograms showed in **2a**. Relative expression values of DMP1- (**a1**), SPP1- (**a2**), BGLAP- (**a3**), and IBSP-mRNA (**a4**), normalized by the amounts of GAPDH-mRNA expression were measured by Gel Doc gel documentation software. The cells were cultured in the absence (−, open bar) or the presence (+, closed bar) of OSC-containing medium. Colums represent mean values of four donors (−) and eight donors (+). **b** Mineralization of human osteoblasts from a primary culture. Scanning electron micrographs showing irregular- and rough cell surface after 15 days of cultivation in OSC-supplemented medium (**b1**) in contrast to osteoblasts growing in medium without OSC-supplementation (**b2**). Phase-contrast microscopic analysis showing calcium deposits (mineralization) in osteoblasts cultivated in OSC supplemented medium over cultivation periods of 5 days (**b3**, **b4**), 10 days (**b5**, **b6**), and 15 days (**b7**, **b8**) respectively. Alizarin-stained cells (**b3**, **b5**, **b7**); not Alizarin-stained cells (**b4**, **b6**, **b8**)

To determine whether OSC (20 μg/ml) stimulates the expression of genes involved in the process of mineralization in osteoblasts, the expression of DMP1-, BGLAP-, SPP1-, and IBSP-mRNA was analyzed by means of RT-PCR after a cultivation period of 15 days. As shown in Fig. 2a an increase in expression level of these genes was detected, compared to non-OSC-treated osteoblasts. DMP1 and SPP1 have proven to be the most strongly up-regulated genes. Their expression increased by 12 times to that of non-OSC-treated osteoblasts (Fig. 2a1, a2), and the expression of BGLAP and IBSP were increased about 8-fold each in contrast to non-OSC-treated osteoblasts (Fig. 2a3, a4).

### Mineral morphology and calcium analysis

**Fig. 3:**
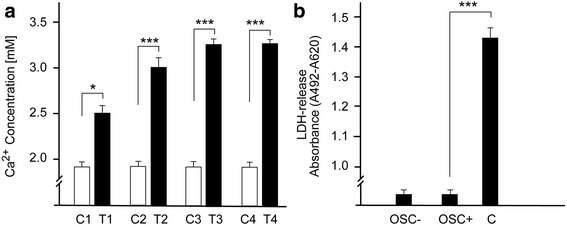
Time course of mineralization. **a** Osteoblasts (2 × 10^6^ each) were grown in MEM supplemented with OSC (20 μg/ml) over a period of 5 days (T1), 10 days (T2), 15 days (T3) and 20 days (T4) in comparison to control cells (C1, C2, C3, C4) grown under identical conditions except for OSC. **b** Measurement of LDH activity. After a 15-day cultivation period of each of 2 × 10^6^ osteoblasts growing in OSC-supplemented media (OSC+) and control osteoblasts growing in non-OSC-supplemented media (OSC-) the LDH activity in the cell free supernatants was determined as described in Material and Methods. Osteoblasts treated with 1% (*v*/v) Triton-×100 for 1 hour (**c**) served as a second control. The experiments were carried out with three primary cultures in triplicate

When examining the mineralization of the osteoblasts in growth medium supplemented with OSC there were qualitative differences when compared to the control cells growing in non-OSC-supplemented medium. As shown in Fig. 2b1, b2 the mineralization process is associated with visible surface roughness of the osteoblasts after cultivation in OSC-supplemented medium for 15 days (Fig. 2b1) which is not recognizable on the surface of osteoblasts cultivated in non-OSC-supplemented medium (Fig. 2b2). To determine whether the specific nodules were mineralized, they were stained with Alizarin red to show formation of calcium deposits. From Fig. 2b3, b5, b7 it is evident that osteoblasts developed distinct mineral nodules over a 5-, 10-, and 15-day-cultivation period in OSC-supplemented media and the nodules increased both in number and size as a function of time. After characterizing the cell surface coverage and calcium deposits, a colorimetric endpoint assay was used to quantify the amount of calcium under all conditions applied. The total calcium content in osteoblasts growing in culture media supplemented with OSC was noticeably higher than in osteoblasts growing in non-OSC-supplemented media, and the calcium content increased with time (Fig. 3a). Supporting the results depicted in Fig. 2b most prominent calcium deposition occurred 15 days (>3 mM) after growing the osteoblasts in culture media supplemented with OSC. Longer incubation time (20 days) did not influence the level of mineralization.

### Cell cytotoxicity assay

To prove whether OSC has an effect on the viability of osteoblasts the LDH cytotoxicity assay was used as cell death marker. A cytotoxic potential of OSC could be assumed if incubation of the cells with OSC results in decreased cell viability. No significant difference was observed in cell vitality and proliferation (as measured by LDH-secretion) between osteoblasts cultivated in OSC-supplemented media over a cultivation period of 15 days versus control osteoblasts growing in the absence of OSC. By comparison, high LDH levels were obtained when cells were treated with the nonionic cell membrane solubilisation detergent triton-×100 for 1 hour (Fig. 3b).

### Analysis of the ERK and AKT pathways

**Fig. 4:**
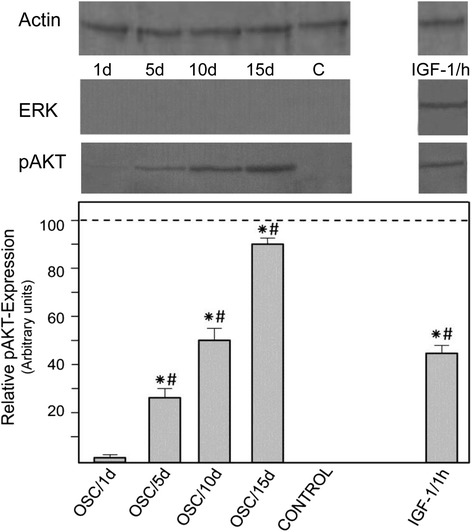
Signaling pathway in osteoblasts. The top panel shows representative Western blots of actin, ERK1/2^PTyr204^, and AKT^PSer473^ . The cells from a primary culture were grown both in OSC-supplemented medium (20 μg/ml) over a period of 1, 5, 10, and 15 days as well as in medium supplemented with 100 ng/ml IGF-1 for 1 h. Osteoblasts growing over the period of 15 days without OSC-supplementation served as control (c). The bottom panel summarizes the results of three densitometric measurements of AKT^Ser473^ expression (grey column) in relation to the expression level of actin (dotted line). Asterisks indicate significant differences when compared to control (*p* < 0.001) und hashtags indicate significant differences when compared to day 1 (p < 0.001)

In order to determine which of the known signal transduction pathways may be involved in the mineralization of primary osteoblasts, we studied the AKT and ERK pathways which are known to promote osteoblast differentiation [25], and can be activated by IGF-1 within 1 h at a concentration of 100 ng/ml [21]. With regard to OSC, we addressed the question whether the AKT and ERK pathways are activated in osteoblasts after their treatment with OSC for 1, 5, 10, and 15 days respectively. Cellular lysates were analyzed by Western blot using phospho-specific antibodies that recognize active ERK or AKT, and actin was used as loading control. The results demonstrate that incubation of the osteoblasts in MEM supplemented with OSC (20 μg/ml) over the above indicated time period had no effect on the ERK pathway as shown by the lack of ERK phosphorylation at tyrosine 204 (Fig.4). However, we found that under the same experimental conditions OSC elicits sustained AKT activation as indicated by AKT phosphoralytion at serine 473, that is hardly detectable at 1 day and continuously increases over the period of 5, 10 and 15 days respectively (*P* < 0.001 Fig. 4). In contrast, no phosphorylation of AKT is detectable in day 15 controls growing under identical conditions except for OSC. We also found that signaling by IGF-1 strongly activates AKT and ERK.

## Discussion

We have carried out a single-center open-label human study to prove the efficacy of OSC for the indications skeletal pain and flexibility. The findings provided a statistical significant positive effect on pain alleviation of 68% after a 20-day-treatment. This study can be considered as a necessary first step in exploring a novel application of intervention, and can be used to evaluate the feasibility and/or modification needed in the design of a large clinical study.

The data presented here also describes the effect of OSC on primary osteoblasts in vitro that exhibit bone tissue specific metabolic responses and produce structural components of bone. The expression of alkaline phosphatase and collagen type-1 in the cytoplasm showed that these cells are of osteoblastic lineage. Although alkaline phosphatase is associated with the osteoblast phenotype, in heavily mineralized cultures cellular levels of this enzyme decline [26]. We studied the expression of bone-specific genes and observed the formation of mineralized nodules when cells were cultured in OSC-supplemented media.

The regulation of bone-specific gene expression reflects the 3 principle periods of osteoblast development: proliferation, extracellular matrix maturation, and mineralization [26]. Here we considered mineralization as functional in vitro endpoint reflecting advanced cell differentiation. Studying the mineralization process we focused on the beginning of cell differentiation. At this period there was no mineral nodule formation as shown by negative Alizarin red staining. Microscopic observation of the mineralization process in osteoblasts showed calcium deposits as an indicator of successful in vitro bone formation over cultivation periods of 5 days. The mineralization process dramatically increased as a function of time indicated by formation of increased numbers of mineralized nodules. In this context, we examined the temporal signaling action of OSC on osteoblast mineralization analyzing ERK and AKT, because it is known that these signal transduction pathways play an important role in osteoblast differentiation [21, 27, 28]. We have shown here that OSC treatment of the osteoblasts does not stimulate ERK activation but induces AKT activation which is associated with enhanced expression of osteoblast-specific genes involved in mineralization. The lack of ERK activity was somewhat unexpected. ERK activity may induce factors required early in the differentiation program as was shown in primary calvarial osteoblasts [21].

The most abundant non-collagenous protein in bone is BGLAP synthesized exclusively by osteoblasts, and is considered to be an indicator of osteoblast differentiation [29]. BGLAP binds to hydroxilapatit and calcium and its expression is modulated by parathyroid hormone [30]**.** The expression of BGLAP suggests that it is involved in the preparation of the extracellular matrix and that the co-expression of alkaline phosphatase may support the onset and progression of extracellular matrix mineralization [26]. In addition to demonstrating the expression and immunocytochemical localization of BGLAP we studied the expression of IBSP, SPP1, and DMP1 genes preferentially expressed in osteoblasts playing important roles in biomineralization. The proteins encoded by these genes belong to the small integrin-binding ligand, *N*-linked glycoprotein (SIBLING) family of proteins and they are believed to play a key role in the process of bone development by facilitating cellular adhesion, mineral nucleation, and mineral maturation [31]. High up-regulation of the SSP1 and DMP1 genes was observed in osteoblasts after an incubation period of 15 days. While the SPP1 gene-encoded protein plays a role in osteoclasts attachment [32] DMP1 and IBSP enhance osteoblast differentiation and are critical components for proper bone mineralization [33, 34]. In this study the appearance of BGLAP, SPP1, DMP1, and IBSP was detected at the same time and their expression coincided with the mineralized nodule formation in osteoblasts. Moreover, nodule formation strongly correlated with elevated calcium emphasizing the importance of calcium in osteoblast mineralization in vitro. Indeed, with the onset of mineralization BGLAP, SPP1, DMP1, and IBSP are induced and their expression is increased in accordance with the accumulation of mineral [26, 34–36]**.**

The biocompatibility of OSC was examined utilizing the LDH cytotoxicity test to study cell growth, reproduction and morphology. Serial and comparative tests, demonstrated that OSC containing culture media had no toxic effect on osteoblasts as seen by cell attachment, growth, with no LDH release into the culture medium or cell destruction.

## Conclusions

In this open-label pilot trial we compared the pain along the treatment in comparison to the initial pain and found that the pain level of the patients treated with OSC considerably decreased (68%) over a treatment period of 20 days. The findings should be interpreted in light of the limitations of this study (i.e. the sample size is modest and no untreated control group was studied). To corroborate the findings further randomized placebo-controlled trial will follow. The in vitro findings provide an insight into the action mechanisms by which OSC is able to contribute to mineralized nodule formation in association with the activation of the AKT signal transduction pathway, which can be involved in the stimulation of specific gene expression required for the susceptibility of primary osteoblasts to bone formation.

## Abbreviations

AKT: Serine/threonine kinase 1
BGLAP: Bone γ-carboxylglutamic acid-containing protein
DMP1: Dentin matrix phosphoprotein
ERK: Extracellular signal-regulated kinase
HMDS: Hexamethyldisilazane
IBSP: Integrin binding sialoprotein
MEM: Minimum essential medium
NPRS: Numeric pain rating scale
OSC: Oscarvit
SDS-PAGE: Sodium dodecyl sulfate polyacrylamide gel electrophoresis
SEM: Scanning electron microscope
SPP1: Secreted phosphoprotein-1
